# Supplementary material for: High-Resolution Linear Epitope Mapping of the Receptor Binding Domain of SARS-CoV-2 Spike Protein in COVID-19 mRNA Vaccine Recipients
Source: Microbiol Spectr. 2021 Nov 10;9(3):e00965-21. doi: 10.1128/Spectrum.00965-21 (PMC8579840; doi:10.1128/Spectrum.00965-21)
Supplement: SUPPLEMENTAL FILE 1 — Supplemental material. Download SPECTRUM00965-21_Supp_1_seq10.pdf, PDF file, 1.1 MB [file spectrum00965-21_supp_1_seq10.pdf]

Supplemental Material

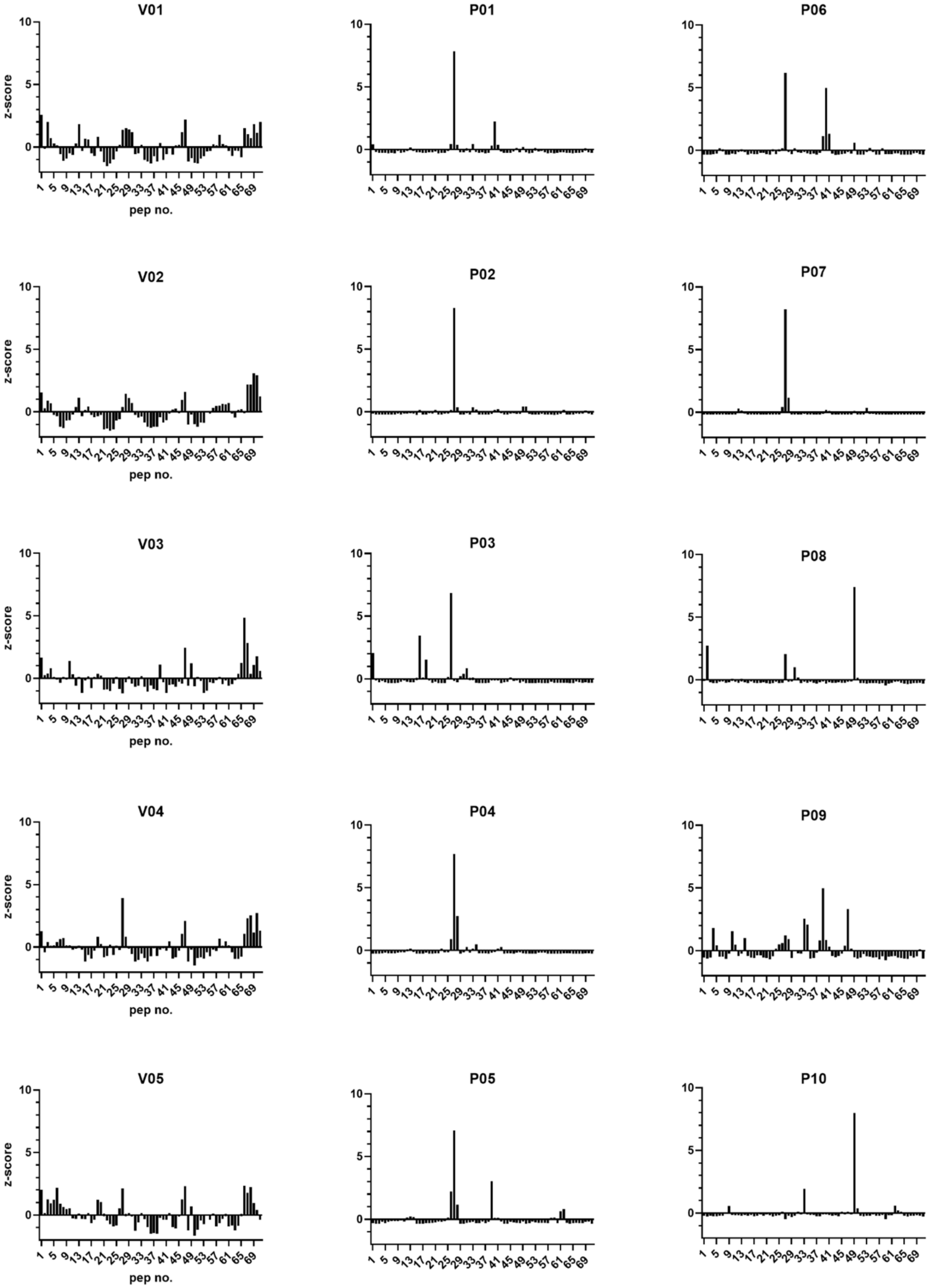

Supplement Figure 1. Epitope profiles of individual subjects are shown in graphs depicting z-scores of each peptide signal, calculated individually, on y-axis, overlapping peptide sequence on x-axis. Vaccine-induced sera (V01–V05) had more variety in recognizing epitopes than infection-induced sera (P01–P10).

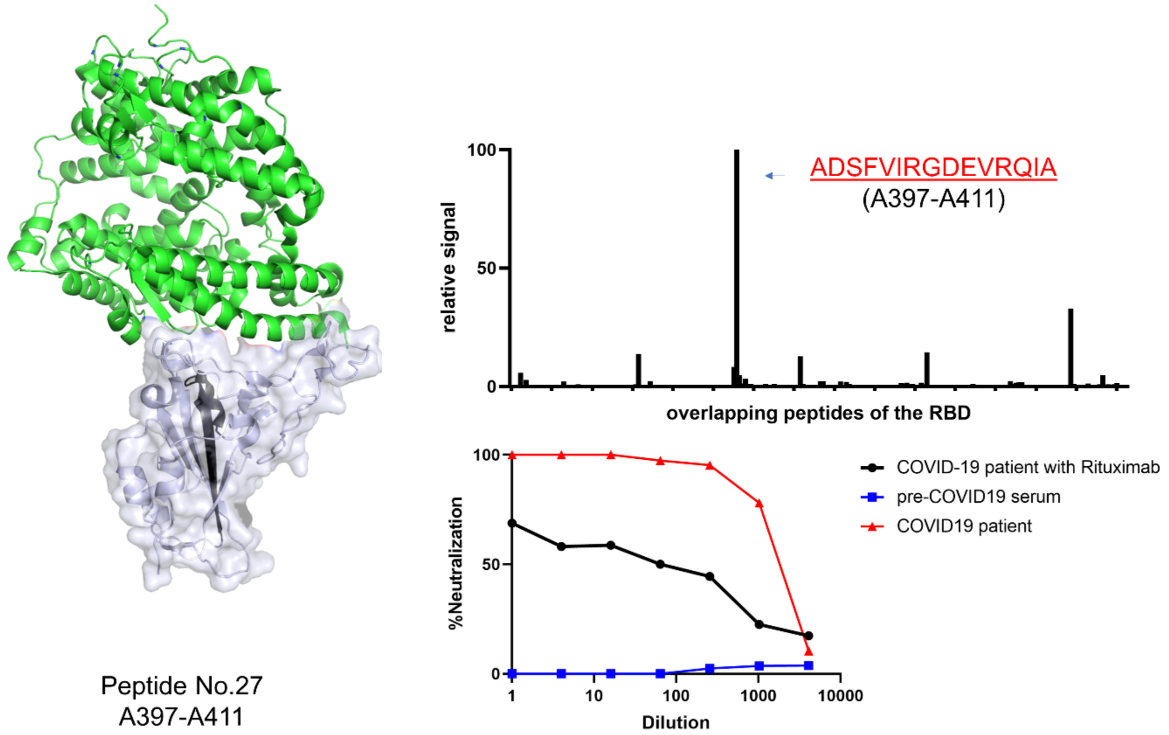

Supplement Figure 2. Epitope profile of a COVID-19 patient who had received Rituximab treatment. The antibodies targeting the peptide No. 27, corresponding to A397–A411 of RBD, were dominant in the epitope profile of this patient (depicted in the upper right graph), which showed limited neutralization compared to a COVID-19 positive serum sample from another COVID-19 patient. Graphic on the left shows the ACE2-RBD complex (ACE2 in green, RBD in tint blue). The position of peptide No.27 is depicted in dark gray.

Supplemental Table 1. Detailed information of subjects from vaccine recipients(N=21) and COVID-19 patients (N=20).

Vaccine recipients	age range	sex	serum collection date after second dose (d)	NT50	anti-RBD IgG	ratio anti-RBD IgG/NT50	anti-N IgG
V01	31-40	M	21	151.1	12630.5	83.6	0.11
V02*1	61-70	M	21	41.0	2829.3	69.0	0.23
V03	31-40	F	21	463.6	31933.3	68.9	0.12
V04	31-40	M	20	246.4	8870.4	36.0	0.06
V05*1	51-60	F	19	235.6	39681.9	168.4	0.05
V06*1	31-40	M	20	222.1	14224.0	64.0	0.06
V07*1	31-40	F	20	360.3	12494.9	34.7	0.03
V08*1	31-40	F	17	515.6	16998.9	33.0	0.40
V09	31-40	F	21	218.8	15608.2	71.3	0.01
V10	41-50	M	21-28	33.8	5234.3	154.8	0.03
V11	41-50	F	21-28	165.7	7298.5	44.0	0.01
V12	31-40	F	21-28	487.9	20656.3	42.3	0.04
V13	51-60	F	21-28	49.3	6136.1	124.6	0.03
V14	51-60	F	21-28	64.9	1485.3	22.9	0.02
V15	51-60	F	21-28	222.9	9074.9	40.7	0.02
V16	41-50	F	21-28	80.8	3484.7	43.2	0.02
V17	31-40	F	21-28	24.2	710.1	29.3	0.04
V18	41-50	F	21-28	539.9	2760.8	5.1	0.01
V19	51-60	F	21-28	239.0	4371.4	18.3	0.01
V20	31-40	F	21-28	149.7	16783.7	112.1	0.03
V21	81-90	F	21-28	225.4	227.6	1.0	0.03

COVID-19 patients	age range	sex	days after onset (d)	NT50	anti-RBD IgG	ratio anti-RBD IgG/NT50	severity of the disease*2
P01*1	51-60	M	19	17414.0	7231.4	0.4	critical
P02*1	31-40	M	12	1902.0	13110.0	6.9	critical
P03*1	71-80	M	20	1449.0	19727.0	13.6	critical
P04*1	41-50	M	18	3271.0	20586.5	6.3	critical
P05*1	61-70	M	29	1029.0	12728.0	12.4	critical
P06*1	81-90	M	16	3959.0	21184.0	5.4	critical
P07*1	71-80	M	20	14990.0	36627.7	2.4	critical
P08*1	21-30	F	14	212.6	1351.7	6.4	mild
P09*1	71-80	M	10	2174.0	3449.9	1.6	moderate
P10*1	31-40	F	13	data missing*3			moderate
P11	21-30	F	49	268.0	1051.5	3.9	mild
P12	51-60	F	49	331.9	4815.4	14.5	moderate
P13	21-30	F	49	176.1	866.7	4.9	mild
P14	21-30	F	49	328.0	1355.8	4.1	mild
P15	21-30	F	63	129.3	1185.2	9.2	mild
P16	31-40	F	49	382.4	2198.7	5.7	moderate
P17	51-60	F	49	374.8	5433.1	14.5	mild
P18	21-30	F	49	208.0	3470.2	16.7	mild
P19	31-40	F	49	154.8	1195.0	7.7	moderate
P20	41-50	F	63	376.6	5069.5	13.5	moderate

*1 Subject included in the epitope analysis

*2 WHO COVID-19 Clinical management: living guidance, 25 January 2021, accessed 30 June 2021

*3 Not enough serum volume could be obtained for these serological markers.

anti-RBD IgG; unit, AU/mL; Abbott SARS-CoV-2 IgG II Quant assay

anti-N IgG; unit, Index; Abbot SARS-CoV-2 IgG assay

Supplemental Table 2. Sequence of the peptides used in this study.

Peptide No.	Sequence
1	RVQPTESIVRFPNIT
2	PTESIVRFPNITNLC
3	SIVRFPNITNLCPFG
4	RFPNITNLCPFGEVF
5	NITNLCPFGEVFNAT
6	NLCPFGEVFNATRFA
7	PFGEVFNATRFASVY
8	EVFNATRFASVYAWN
9	NATRFASVYAWNRKR
10	RFASVYAWNRKRISN
11	SVYAWNRKRISNCVA
12	AWNRKRISNCVADYS
13	RKRISNCVADYSVLY
14	ISNCVADYSVLYNSA
15	CVADYSVLYNSASFS
16	DYSVLYNSASFSTFK
17	VLYNSASFSTFKCYG
18	NSASFSTFKCYGVSP
19	SFSTFKCYGVSPTKL
20	TFKCYGVSPTKLNDL
21	CYGVSPTKLNDLCFT
22	VSPTKLNDLCFTNVY
23	TKLNDLCFTNVYADS
24	NDLCFTNVYADSFVI
25	CFTNVYADSFVIRGD
26	NVYADSFVIRGDEVR
27	ADSFVIRGDEVRQIA
28	FVIRGDEVRQIAPGQ
29	RGDEVRQIAPGQTGK
30	EVRQIAPGQTGKIAD
31	QIAPGQTGKIADYNY
32	PGQTGKIADYNYKLP
33	TGKIADYNYKLPDDF
34	IADYNYKLPDDFTGC
35	YNYKLPDDFTGCVIA
36	KLPDDFTGCVIAWNS
37	DDFTGCVIAWNSNNL
38	TGCVIAWNSNNLDSK
39	VIAWNSNNLDSKVGG
40	WNSNNLDSKVGGNYN
41	NNLDSKVGGNYNYLY
42	DSKVGGNYNYLYRLF
43	VGGNYNYLYRLFRKS
44	NYNYLYRLFRKSNLK
45	YLYRLFRKSNLKPFE
46	RLFRKSNLKPFERDI
47	RKSNLKPFERDISTE
48	NLKPFERDISTEIYQ
49	PFERDISTEIYQAGS
50	RDISTEIYQAGSTPC
51	STEIYQAGSTPCNGV
52	IYQAGSTPCNGVEGF
53	AGSTPCNGVEGFNCY
54	TPCNGVEGFNCYFPL
55	NGVEGFNCYFPLQSY
56	EGFNCYFPLQSYGFQ
57	NCYFPLQSYGFQPTN
58	FPLQSYGFQPTNGVG
59	QSYGFQPTNGVGYQP
60	GFQPTNGVGYQPYRV
61	PTNGVGYQPYRVVVL
62	GVGYQPYRVVVLSFE
63	YQPYRVVVLSFELLH
64	YRVVVLSFELLHAPA
65	VVLSFELLHAPATVC
66	SFELLHAPATVCGPK
67	LLHAPATVCGPKKST
68	APATVCGPKKSTNLV
69	TVCGPKKSTNLVKNK
70	GPKKSTNLVKNKCVN
71	PKKSTNLVKNKCVNF
Single mutation containing peptides	
	K417N
29	RGDEVRQIAPGQTGN
30	EVRQIAPGQTGNIAD
31	QIAPGQTGNIADYNY
32	PGQTGNIADYNYKLP
33	TGNIADYNYKLPDDF
	K417T
29	RGDEVRQIAPGQTGT
30	EVRQIAPGQTGTIAD
31	QIAPGQTGTIADYNY
32	PGQTGTIADYNYKLP
33	TGTIADYNYKLPDDF
	E484K
52	IYQAGSTPCNGVKGF
53	AGSTPCNGVKGFNCY
54	TPCNGVKGFNCYFPL
55	NGVKGFNCYFPLQSY
56	KGFNCYFPLQSYGFQ
	N501Y
57	NCYFPLQSYGFQPTY
58	FPLQSYGFQPTYGVG
59	QSYGFQPTYGVGYQP
60	GFQPTYGVGYQPYRV
61	PTYGVGYQPYRVVVL

Supplemental Table 3. Raw signal intensities (blank signals are subtracted).

raw-bl	V02	V05	V06	V07	V08	P01	P02	P03	P04	P05	P06	P07	P08	P09	P10
1	5898.755	11736.7	3502.173	4159.933	16225.4	1051.194	59.02	2425.419	1.929	176.222	131.111	-4.778	41.656	15.889	4.333
2	1350.664	6681.4	1729.264	1373.111	7120.8	162.031	12.223	238.667	-5.06	54.445	96.267	-4.222	584.513	8.889	-2.889
3	4975.265	8967.7	1907.9	2725.666	12677.1	25.767	16.111	83.222	11.495	88.778	135.322	0.889	17.677	14.666	2.889
4	2754.3	8186.6	2428.627	1970.777	11423.3	32.011	15.667	209.667	121.374	603.223	283.856	-3.888	5.283	324.556	-0.222
5	2092.24	4444.8	1547.809	2309.733	12655	22.02	18.555	69.475	232.293	156.667	341.511	-6.111	8.475	146.361	-1.333
6	1549.664	4037.4	1515.173	2754.444	16892.9	15.262	23	27.989	121.989	538.111	1184.744	-3.334	24.899	27.444	1.333
7	640.8456	760.8	987.7179	3153.969	11193.9	43.949	23.889	12.495	150.677	1520.889	946.422	-0.556	67.586	26	4.889
8	-241.063	346.4	1326.173	3226.666	10198.6	11.798	8.667	16.616	126.707	795.222	152.189	14	12.071	3.222	25.889
9	75.29957	2671.5	1294.355	2029.111	9290.7	230.273	50.333	43.636	229.1	803.667	213	110.556	19.636	59.333	84.333
10	783.6636	2775.9	3155.627	2308.111	9684.2	48.717	11	115.136	421.444	986.444	345.044	79.333	77.808	292.444	30.777
11	565.5726	4727.5	1831.082	1770	6061.6	70.353	56.777	223.829	442.262	1359.444	273.744	101.777	26.171	150.444	7.888
12	2086.573	7081.68	668.8999	1886.333	5961.1	521.278	50.778	111.596	1345.869	1190.111	1404.378	1720.778	15.869	34.111	7.111
13	4647.755	9963.40	1550.718	2269.444	7744.2	655.762	77.889	81.697	862.083	2012.333	1528.19	554.333	37.904	57.111	4.889
14	1086.573	4081.68	-57.0091	1769.889	5955.4	509.142	54.889	78.596	1265.323	1897.445	797.378	291	20.596	218.889	8.334
15	2692.3	5300.4	1196.718	220.3333	5703.7	78	2.111	258.969	10.515	104.444	131.333	-6.667	4.606	36.444	2.777
16	2582.3	7162.1	1384.446	1027.777	7176.4	72.678	126.861	3853.064	35.178	73.445	455.667	12.889	33.678	15.778	8.778
17	790.2726	4547.9	453.0819	586.6663	4360.5	18.856	3.778	496.402	38.778	39.112	235	-4.111	10.829	10.334	-0.444
18	440.4816	3810.4	1532.627	1619.333	5671.8	33.478	6.111	1892.547	321.945	195.222	310.978	-0.111	5.233	39.945	4.222
19	2945.573	4096.4	1838.809	3424.333	12593.7	75.333	83.222	239.717	105.844	275	615.044	6.333	11.535	39.694	12.694
20	1009.3	4528.9	1680.264	2497.444	11885.3	64.411	62.667	35.929	105.986	310.333	672.411	0.667	22.111	17.555	3.555
21	-413.245	-156.5	273.2639	793.2223	6931.4	166.677	112.639	109.222	128.858	413.389	241.822	4.333	7.586	11.889	13.444
22	-913.245	184.6	306.8999	921.8893	5217.6	5.811	5.778	5.747	112.475	745.528	101.411	-0.556	-1.434	-1.778	7.667
23	-590.245	-556.1	115.4459	2383.222	4072.1	17.678	13.667	1.687	859.349	525.556	932.361	71.556	3.869	32.111	-2.778
24	-22.0634	-210.1	890.2639	1027.889	3273.6	44.011	56.333	24.566	481.194	835.361	319.611	12.222	26.566	106.778	2.222
25	1029.664	2737.1	1561.809	1963	3496.4	241.1	50.778	319.909	409.273	1208	939.778	88.444	7.545	155.889	7
26	1917.823	3163.8	335.1729	1712.777	9629.1	1082.079	235.666	7295.389	4525.586	9002.068	1748.922	2271	16.313	168.222	16.778
27	3878.406	7128.6	-70.4641	8509.976	16659.01	12103.16	5406.706	370	30378.3	25759.35	21583	30366.44	455.067	247.111	-28.556
28	4155.073	11292.5	1004.536	3399.111	7624.8	1007.578	368.667	70.596	11428.14	5326.889	1499.278	4860.111	29.414	207.778	1.556
29	3932.755	9894.7	1594.718	2182.788	7861.5	66.844	7.333	595.717	34.899	89.555	252.544	7.333	34.044	12.555	-7
30	3599.3	8319.0	893.6269	1149.555	7451.009	100.589	25.333	776.162	1113.356	118.667	1204.525	52.043	253.089	88.556	4.445
31	674.0276	4485.7	583.0819	160.0003	1701.1	543.361	81.384	1247.02	2065.911	300.111	1364.838	85.202	94.211	62	18.111
32	767.6636	3662.1	673.4459	432.0003	4726.2	88.678	15.445	248.323	365.778	482.111	588.778	16.667	8.878	60	14

33	1575.209	3954.1	1644.718	1247	7179.4	1100.692	363.214	283.909	797.462	574.778	1407.636	797.643	60.6	421.917	230.643
34	-123.427	2180.4	565.0819	626.4443	5718.4	135.078	265.695	55.233	2872.153	166.222	466.978	69.334	16.078	362.086	13.667
35	-348.154	751.2	96.0819	179.6663	2939.4	36.944	27.222	13.262	210.08	216.888	292.694	-6.445	25.319	7.555	11.777
36	-590.609	470.1	711.8999	883.5553	587.5	81.322	23.889	7.131	143.805	735.222	448.922	-0.778	10.122	13.778	12.111
37	361.2086	640.3	352.6269	1940	888.6	9.789	7.667	14.253	139.778	157.667	171.589	12.778	-2.011	68.778	-1.667
38	-312.7	683.2	218.1729	913.2223	613.3	39.689	6.778	62.525	95.389	524	572.289	9.889	21.989	194.333	-0.111
39	2157.573	3845.4	2801.173	1727.889	6165.8	854.889	34.556	421.556	267.465	11821.13	5040.973	152.181	33.356	738.156	31.556
40	-109.7	2167.4	1010.082	2234.777	5528	3764.19	125.889	414.583	1179.969	1635.333	17682.9	663.889	-0.167	203.111	13.444
41	632.4816	2833.8	-50.9181	1625.333	5622.1	961.653	296.378	239.778	764.278	1108.445	5588.687	1076.978	19.078	133.111	11
42	1744.482	4911.9	765.4459	2842.555	7189.9	187.233	88.333	28.424	2123.878	642.111	353.533	740.444	11.133	34.555	1.889
43	582.8456	6257.2	796.4459	574.8893	2949.8	17.433	6.777	72.433	40.242	78.555	87.333	-1.556	11.333	19.444	5.777
44	1549.3	6526.9	572.8999	760.4443	2503.8	16.767	10.778	119.367	130.042	112.223	221.334	0.667	22.567	36.445	0.556
45	1567.209	5797.1	1088.809	1602.889	7512.4	36.236	63.778	289.911	128.222	526.778	279.411	-3.111	10.911	59.444	18.333
46	3622.573	9249.8	883.4459	3854.788	12727.46	167.933	86.151	388.333	85.424	435.666	411.633	35.222	13.933	138.111	14.111
47	5220.028	11884.5	4496.173	5500.555	17363.4	357.542	44.81	221.867	333.167	304.445	1420.834	58.238	27.667	523.205	18
48	-353.882	1395.7	640.7179	211.5553	1770.2	99.144	38.555	47.144	379.888	242.111	390.844	5.444	34.944	86.555	14
49	162.7546	4700.1	2929.264	1724.151	10311.1	711.136	427.944	171.027	144.444	722.535	3246.301	470.829	1496.044	21.555	873.194
50	-516.609	1492.7	618.7179	-339.667	0	64.022	421.355	15.622	70.722	137.778	222.122	31.555	50.222	8.889	66.555
51	-553.609	754.5	1319.627	625.7773	1996	9.944	42	11.544	68.171	189.777	113.544	21.555	8.044	19	4.333
52	80.14357	2135.4	1138.809	783.4443	5376.1	30.022	25.555	26.422	36.313	660.444	160.622	102.778	4.522	55.333	-1.778
53	377.6636	2037.9	-38.8271	593.7773	4025.1	324.653	7.667	21.378	84.869	485.889	864.334	1952.578	-2.522	29.207	3.889
54	1030.209	5487.6	193.5359	1436.889	7255.4	188.556	24.667	45.578	224.323	346.111	1860.111	168.334	5.078	24.778	3.889
55	1060.028	5634.9	1004.991	849.4443	5694.8	238.333	35.556	30.4	278.833	316.667	905.1	152.889	9.3	17.222	14.222
56	1939.482	6718.9	936.4459	1728.666	6976.6	86.133	14.889	13.833	303.333	366.555	183.333	82.777	6.633	20.444	2.111
57	1858.755	7426.6	1230.446	1594.444	3266.7	15.178	7.778	21.978	4.051	216.445	103.678	-8.111	2.278	-0.444	3.667
58	3233.028	7517.6	1334.718	3192.222	4397.555	17.522	21.444	122.522	17.222	1050.949	1164.858	1.222	6.022	28.889	8.778
59	1983.755	8075.6	852.2639	1949.333	5918.2	-11.333	-2.666	39.167	62.212	1184.485	242.889	55.667	-27.633	-8.889	-26.889
60	1525.209	7869.0	1421.536	2826	6767.5	43.944	16.111	45.666	91.353	213.222	232.144	4.555	2.744	26.444	7.444
61	1095.3	8340.6	648.5359	2039.444	3357.3	107.856	37.667	54.156	74.101	3454.623	228.985	-0.222	15.556	31.223	6.889
62	445.4816	5717.5	814.7179	1379.333	3560.2	73.822	130.555	41.922	150.767	4060.495	534.222	0.666	23.722	35.555	87.778
63	1116.209	3735.2	1487.627	555.7773	1898.6	50.222	21.333	51.347	114.022	279.222	475.936	24.597	34.793	17.222	47.055
64	1059.3	5344.5	1845.809	516.4443	3412.8	27.878	14.778	25.178	109.653	130.556	180.378	10.778	12.178	11.111	18.667
65	261.1176	6324.9	2961.082	846.0003	7649.3	7.433	12.111	8.633	60.904	196.333	132.133	-1.667	0.533	9.555	3.889
66	4106.973	5840.6	7496.991	3843.222	17577.37	39.233	52.666	158.633	73.878	361.555	139.133	10.555	-5.467	2.222	-5.556

67	3310.573	14211.8	4998.355	5835.111	15127.4	44.056	42.223	65.256	83.011	163.667	222.856	67	-1.344	34.889	1.112
68	2791.664	14216.4	1839.991	6243.06	17157.37	86.433	52.666	39.133	90.788	196.111	534.333	38.777	3.933	16.222	2.222
69	4669.573	17842.9	2764.082	4012	11441.7	297.667	94.223	93.267	115.849	592	566.267	33.778	8.667	29.223	6.556
70	3448.482	17271.2	3651.991	6563.633	8992.2	97.078	50.153	48.578	163.323	727.778	231.178	19.556	13.178	104.921	3.778
71	4970.937	10457.1	2181.082	4219.666	5655.6	18.364	16.222	36.4	21.364	134.889	95.727	-2.556	3.2	8.111	-3.111
K417N															
29	4217.118	10766.68	2788.627	2386.111	8558.017	48.142	40	52.978	130.051	194.556	871.778	9.222	7.478	52.667	0.222
30	3562.664	13991.4	2955.173	6844.666	11278.93	28.525	54.222	156.289	188.162	341.445	341.889	291.756	-2.611	50.111	6.111
31	761.7546	8268.675	1232.173	3159.444	3155.1	64.707	290	54.589	380.351	676.222	378.98	28.333	11.489	47.222	41.667
32	1325.482	6500.22	3253.536	3263.889	7501.4	651.644	465.361	344.277	542.08	582	511.353	108.544	399.829	72.111	65.555
33	1867.118	10858.58	3653.809	3548.444	6614.3	268.264	407.434	117.889	690.822	340.445	331.344	5.778	23.789	120.735	34.556
K417T															
29	2757.573	7307.402	3687.718	3254.111	11435.4	62.485	109.223	56.167	55.303	517.556	478.122	8.556	7.867	21.334	12.445
30	1084.573	17339.77	9771.173	4062.222	14326.6	25.818	75.222	35.9	168.545	626.778	225.727	24.111	6.6	166.429	20.667
31	858.3906	7182.493	2483.355	3178.889	10349.7	55.838	404.611	22.011	123.929	1206	291.747	24	17.611	82.656	9.444
32	2472.664	7329.675	1902.627	2668.444	11144.1	373.889	1923.197	180.289	234.162	1880.778	964.616	35.445	147.972	82.556	5.889
33	2182.118	15713.04	4810.991	6492.444	8218.4	432.587	662.598	51.044	487.301	2005.666	459.808	6.555	42.744	516.675	0.222
E484K															
52	2924.846	9065.675	2575.355	3591.333	16943.6	83.08	50.888	32.544	40.626	383.444	105.044	-8.667	-2.056	-8.778	2.333
53	2507.209	7142.402	2184.9	3950.444	12114.1	38.889	77.889	1061.489	19.707	221.445	132.489	12.389	-0.011	12.667	15.222
54	3428.118	7688.13	4884.991	3585.444	9925.6	55.273	252.556	68.9	53	347.111	205.5	2	11.1	6.222	9.111
55	3128.937	8151.22	4931.173	4643.889	12089.6	97.079	206.666	43.222	107.131	421.222	104.522	-1.222	43.079	11.555	-0.111
56	2383.391	8093.039	4252.355	4041.111	11561.4	148.445	139.889	33.278	64.403	760.111	152.878	-2.889	15.178	16.889	2.889
N501Y															
57	2262.209	7069.948	5450.264	3454.555	13795.2	135.292	159.467	11.967	39.167	576.778	224.567	-8.111	-1.333	3	-1.666
58	2989.573	7605.311	4383.627	3898.222	9998.8	122.051	247.978	45.445	56.678	525.111	626.028	0.222	9.278	10.222	11.222
59	3274.482	5450.402	3166.446	3356.333	13039.2	99.778	222.078	25.978	101.505	688.334	390.378	8.889	2.478	42.889	37.445
60	2696.209	7658.039	3545.264	4425.444	17699.2	59.929	136.333	31.311	64.747	935.444	200.411	-3.667	1.911	95.111	4.667
61	1722.846	7234.948	4839.718	4558.111	10792.3	116.04	200.555	28.422	117.04	4042.389	187.122	0.111	8.522	38.111	-2.334
